# Loss of TaIRX9b gene function in wheat decreases chain length and amount of arabinoxylan in grain but increases cross‐linking

**DOI:** 10.1111/pbi.13393

**Published:** 2020-05-17

**Authors:** Till K. Pellny, Archana Patil, Abigail J. Wood, Jackie Freeman, Kirstie Halsey, Amy Plummer, Ondrej Kosik, Henry Temple, Joel D. Collins, Paul Dupree, Simon Berry, Peter R. Shewry, Alison Lovegrove, Andrew L. Phillips, Rowan A.C. Mitchell

**Affiliations:** ^1^ Plant Sciences Rothamsted Research Harpenden UK; ^2^ Biochemistry Department University of Cambridge Cambridge UK; ^3^ Limagrain (UK) Ltd Bury St. Edmunds UK

**Keywords:** wheat grain viscosity, IRX9, xylan, ferulic acid, cell wall integrity

## Abstract

Wheat contains abundant xylan in cell walls of all tissues, but in endosperm, there is an unusual form of xylan substituted only by arabinose (arabinoxylan; AX) that has long chains and low levels of feruloylation, a fraction of which is extractable in water (WE‐AX). WE‐AX acts as soluble dietary fibre but also gives rise to viscous extracts from grain, a detrimental trait for some non‐food uses of wheat. Here, we show that a glycosyl transferase family 43 wheat gene abundantly expressed in endosperm complements the Arabidopsis *irx9* mutant and so name the three homoeologous genes TaIRX9b. We generated wheat lines with a constitutive knockout of TaIRX9b by stacking loss‐of‐function alleles for these homeologues from a mutagenized hexaploid wheat population resulting in decreases in grain extract viscosity of 50%–80%. The amount and chain length of WE‐AX molecules from grain of these triple‐stack lines was decreased accounting for the changes in extract viscosity. Imaging of immature wheat grain sections of triple‐stacks showed abolition of immunolabelling in endosperm with LM11 antibody that recognizes epitopes in AX, but also showed apparently normal cell size and shape in all cell types, including endosperm. We identified differentially expressed genes from endosperm of triple‐stacks suggesting that compensatory changes occur to maintain this endosperm cell wall integrity. Consistent with this, we observed increased ferulate dimerization and increased cross‐linking of WE‐AX molecules in triple‐stacks. These novel wheat lines lacking functional TaIRX9b therefore provide insight into control of wheat endosperm cell walls.

## Introduction

Xylan is a major component of primary and secondary cell walls in grasses, typically accounting for 25%–30% of cell wall by dry weight. It exists mostly in highly decorated forms called heteroxylans that have frequent arabinofuranosyl (Araf), glucuronosyl and acetyl substitutions (Scheller and Ulvskov, 2010). Some of these Araf are themselves linked to hydroxycinnamic acids ferulate (FA) and para‐coumarate (pCA). FA is key to grass cell wall function, allowing cross‐linking between chains of xylan and from xylan to lignin via radical coupling of the FA while the role of pCA on xylan is less clear as it does not seem to form cross‐links, but it may be involved in facilitating the radical coupling of FA and of lignin (Ralph, 2010). In wheat endosperm, the tissue of major importance for wheat end uses, cell walls have an unusual composition that is dominated (60%–70% of the polysaccharide) by a simple xylan that has only Araf decorations (arabinoxylan; AX) some of which are feruloylated. A portion (typically 20%–30%) of this endosperm AX is extractable in water (WE‐AX); compared to the rest of endosperm AX (water‐unextractable AX; WU‐AX), WE‐AX has low feruloylation but long chains (Saulnier *et al.*, 2007). The greater solubility of WE‐AX compared to WU‐AX has been attributed to the much lower amounts of diferulate dimers that can act as cross‐links between AX chains (Saulnier *et al.*, 2007). The abundance and long chains of WE‐AX confer viscosity to aqueous extracts of wheat grain, a property that is detrimental for monogastric animal nutrition (Annison, 1991) and for alcohol production. Conversely, wheat grain WE‐AX is an important source of soluble fibre in the human diet (Gebruers *et al.*, 2008); these conflicting needs may lead to an increasing separation between wheat varieties intended for human food and other uses.

The xylan backbone is considered to be synthesized by a complex comprising IRX9, IRX14 and IRX10 proteins (Zeng *et al.*, 2016); IRX9 and IRX14 are encoded by genes in the glycosyl transferase family (GT) 43 and IRX10, which is the catalytically active component (Jensen *et al.*, 2014; Urbanowicz *et al.*, 2014), by GT47 genes. Loss‐of‐function mutations in any of these components in Arabidopsis result in the *irregular xylem* (*irx*) phenotype accompanied by severe dwarfing due to loss of mechanical strength in cell walls. The function(s) of IRX9 and IRX14 in the xylan synthase are not clear but are most likely accessory proteins. The most abundantly expressed genes in wheat endosperm that resemble IRX9 genes are three homeologues that we called TaGT43_2A, TaGT43_2B, TaGT43_2D (Pellny *et al.*, 2012). We have previously shown that transgenic wheat expressing an RNAi construct targeting these genes resulted in decreases in the amount and chain length of WE‐AX in endosperm (Lovegrove *et al.*, 2013) and in decreased extract viscosity (Freeman *et al.*, 2016). Suppression of TaGT43_2 genes did not only affect WE‐AX; the amount of endosperm WU‐AX was also decreased in amount and chain length (Freeman *et al.*, 2016) and while the wall thickness of endosperm cells was decreased, endosperm cell shape was unaltered (Lovegrove *et al.*, 2013). This indicates that endosperm cell wall strength was maintained, likely made possible by high levels of diferulate dimers in the remaining AX such that diferulate per unit endosperm tissue was not decreased in the RNAi lines (Freeman *et al.*, 2017). Effects in these TaGT43_2 RNAi lines would be dictated by spatial and temporal expression of the transgene which was driven by the HMW1Dx5 endosperm‐specific promoter; it was unclear whether a constitutive removal of TaGT43_2 function would have similar effect enabling us to eliminate residue viscosity derived from grain WE‐AX without affecting cell wall function in endosperm and other tissues.

**Figure 1:**
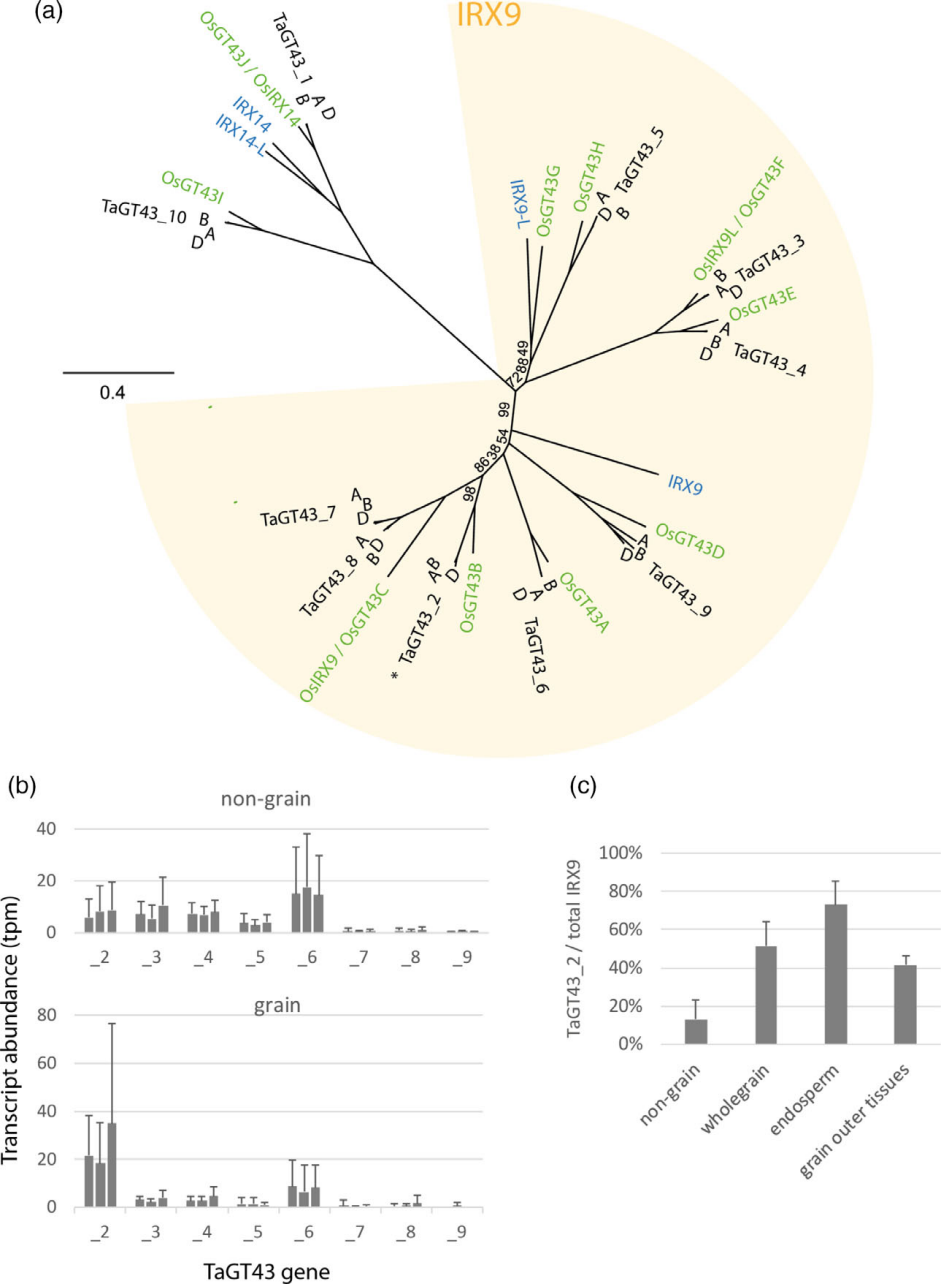
GT43 gene family phylogeny and expression in wheat. (a) Maximum likelihood tree of Arabidopsis (blue text), rice (green) and wheat (black) genes. All wheat genes cluster together as triads from the A, B and D subgenomes and are named as previously (Pellny *et al.*, 2012). Rice genes are as named in Chiniquy *et al. *(2013) and Lee *et al. *(2014). Locus IDs for these genes are given in Table S1. The IRX9 clade in which genes are putatively redundant with Arabidopsis IRX9 is indicated; OsIRX9, OsIRX9L (Chiniquy *et al.*, 2013) and OsGT43A and OsGT43E (Lee *et al.*, 2014) functionally complement *irx9*. (b, c) Public expression data for wheat genes in IRX9 clade taken from the expVIP platform (Borrill *et al.*, 2016); all hexaploid wheat studies were used, using means for each study, source tissue, stage, treatment combination as data points. (b) Transcript abundance (TPM; transcript reads per million mapped reads) of all putative wheat IRX9 genes shown as triads with homeologues ordered A, B, D. Means ± SD for samples from non‐grain (*n* = 134) and grain (*n* = 34) tissues. (c) Proportion of total transcript counts of wheat IRX9 genes accounted for by sum of TaGT43_2A, B and D. Means ± SD for samples from non‐grain (*n* = 134), whole grain (*n* = 13), endosperm (includes starchy endosperm and aleurone) (*n* = 16) and grain outer tissues (*n* = 4).

There are eight triads of homeologous genes resembling IRX9 in wheat (Figure 1a), and the homeologues of each triad have similar expression patterns, as is the case for most wheat triads (Ramírez‐González *et al.*, 2018). While the TaGT43_2 genes are the most highly expressed of these genes in grain, in all other tissues they are less highly expressed and TaGT43_6 tends to be most expressed (Figure 1b). TaGT43_2 transcripts account for ~70% of total putative IRX9 transcripts in endosperm but ~15% in non‐grain tissues (Figure 1c). We therefore postulated that constitutive knockout of TaGT43_2 function would have similar effects to the endosperm‐specific silencing, a decrease in long‐chain WE‐AX from grain, but with no consequences for cell wall function in endosperm or other tissues. Approaches for finding mutations in specific genes of wheat were therefore developed using variants of TILLING methodology on EMS‐mutagenized populations of wheat, cv. Cadenza (Botticella *et al.*, 2011) followed by crossing to achieve complete functional knockouts of gene function. Here, we report use of this approach to develop triple mutants lacking functional TaGT43_2 genes and consequent loss of long‐chain WE‐AX; we also examine pleiotropic effects on the transcriptome and AX molecule cross‐linking.

## Results

### Gene identification

We assessed the likelihood that constitutive knockouts of TaGT43 genes would have effects on cell wall function in tissues other than grain using sequence and expression evidence. TaGT43_2 genes are orthologs of the rice gene OsGT43B which complements the irx9 Arabidopsis mutant (Lee *et al.*, 2014), one of four rice genes that have been shown to complement *irx9* (Chiniquy *et al.*, 2013; Lee *et al.*, 2014). In wheat, there are 8 triads in the same clade as these genes and so seem likely to have IRX9 function, rather than the IRX14 which are also encoded by GT43 genes (Figure 1a). If these genes are indeed functionally redundant, then the transcript abundance evidence (Figure 1c) suggests that a complete knockout of TaGT43_2 function should have little effect in tissues other than grain.

### TaGT43_2 functionally complements IRX9 gene in Arabidopsis

**Figure 2:**
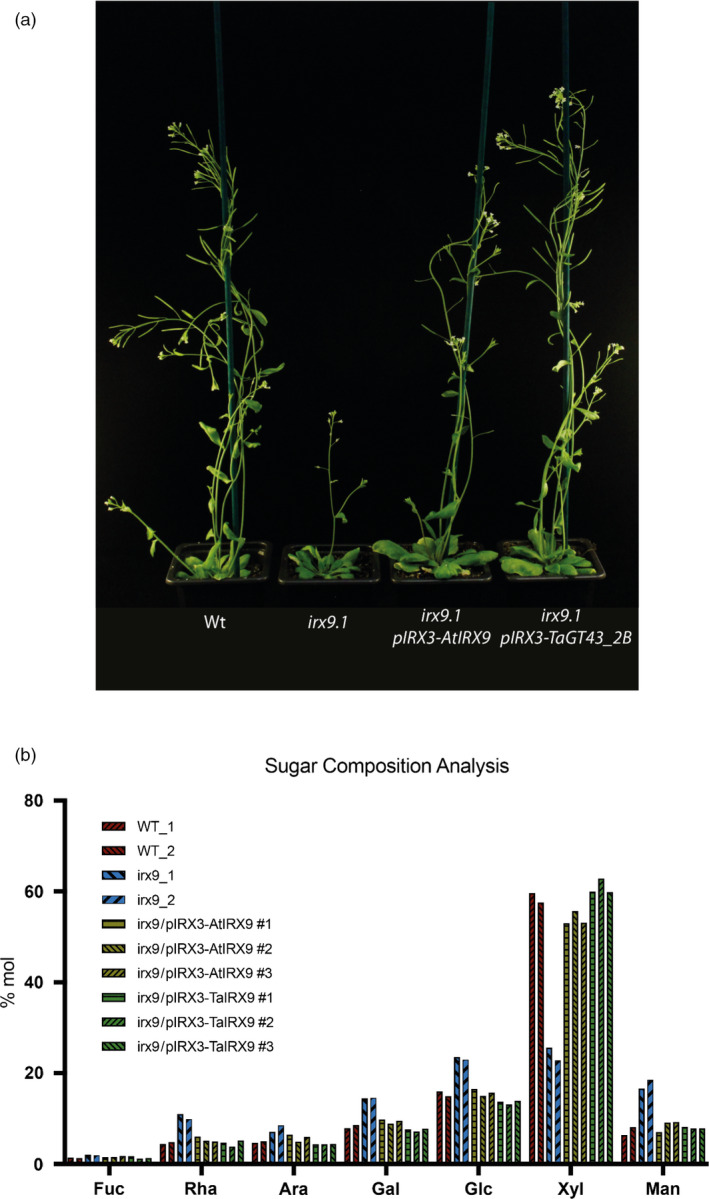
Visual phenotype (a) and (b) neutral monosaccharide composition of AIR from stems of Arabidopsis wild‐type (Wt), *irx9* mutant, *irx9* mutant expressing AtIRX9 gene, *irx9* mutant expressing TaGT43_2B gene under IRX3 promoter, two independent measurements are shown for WT and *irx9* mutant, and three independent lines are measured for complemented *irx9* transgenic plants. Ara, arabinose; Fuc, fucose; Gal, galactose; Glc, glucose; Man, mannose; Rha, rhamnose; Xyl, xylose.

We expressed the coding region of the wheat gene TaGT43_2B driven by the Arabidopsis IRX3 promoter in the Arabidopsis *irx9* mutant to test complementation of knockout of Arabidopsis IRX9. The IRX3 promoter was selected as IRX3 and IRX9 genes have similar expression patterns in Arabidopsis, predominantly in cells depositing secondary cell walls, but IRX3 is preferred over IRX9 due to higher absolute expression. We observed a rescued phenotype in these plants similar to wild‐type and *irx9* lines overexpressing Arabidopsis IRX9 (Figure 2a). We also found that xylose (Xyl) content of alcohol insoluble residue (AIR) was restored to wild‐type levels in *irx9* lines overexpressing Arabidopsis IRX9 or TaGT43_2B (Figure 2b). TaGT43_2 therefore complements *irx9* and we rename this triad TaIRX9b; the b is to reflect the existing nomenclature for rice ortholog OsGT43B (Lee *et al.*, 2014) but using lowercase to avoid confusion with labelling of wheat A, B, D homeologs.

### Identification of mutants

We searched for loss‐of‐function alleles for the TaIRX9b genes in the mutagenized population of hexaploid wheat lines derived from cv. Cadenza (Rakszegi *et al.*, 2010). We used high‐resolution melting to detect SNPs in amplicons produced by nested PCR as described by (Botticella *et al.*, 2011) for starch branching enzyme IIα target wheat genes. We selected mutations that introduced premature stop codons in each of three homeologs of TaIRX9b that were predicted to truncate the encoded proteins by 306, 69, 62 residues for TaIRX9b A, B, D respectively (Figure S1). Co‐dominant competitive allele‐specific PCR (KASP) markers were designed for each of these alleles to facilitate genotyping of progeny from crosses.

### Triple mutants of TaIRX9b knockout homeoalleles confer greatly decreased WE‐AX

**Figure 3:**
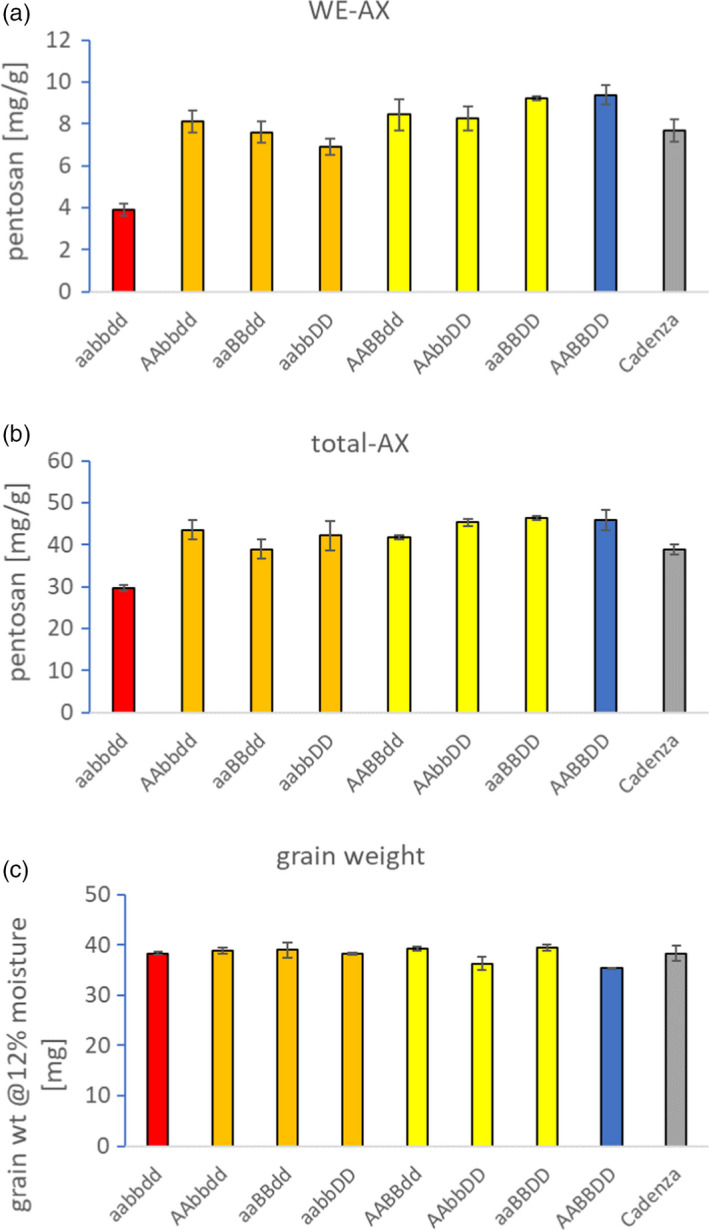
Effect of different stacks of TaIRX9b KO alleles on (a) WE‐AX, (b) total AX from grain and (c) grain weight. In mutant lines and null segregants, allele composition is shown with letter indicating subgenome and lowercase denoting recessive KO allele. Cadenza is non‐mutagenized cultivar. Error bars are ±SE, *n* = 3 independent biological reps.

We compared grain WE‐AX content in lines that were homozygous for these knockout (KO) alleles (Figure 3); lines carrying only one KO homeoallele did not differ significantly from a null‐segregant control line, but one out of the three double stacks (aabbDD) had significantly (*F*‐test, 1‐way ANOVA; *P* < 0.05) lower WE‐AX and the aabbdd triple‐stack had WE‐AX content that was approximately 40% that of control (*P* < 0.001; Figure 3a). This dose effect on WE‐AX of stacking KO alleles is similar to other examples for wheat where a big effect is only achieved when all KO homeoalleles are combined and has been termed the ‘non‐additive dose’ case, which along with the ‘fully redundant’ case reflect hidden variation that would not be found by conventional wheat breeding (Borrill *et al.*, 2019). Total AX content of grain was also decreased in triple mutants to about 65% of null‐segregant control (*P* < 0.001; Figure 3b). There were no significant differences between lines in grain weight (Figure 3c; *F*‐test, 1‐way ANOVA; *P* = 2.41).

### Grain sections from triple mutants show loss of LM11 labelling but apparent normal cell shape

**Figure 4:**
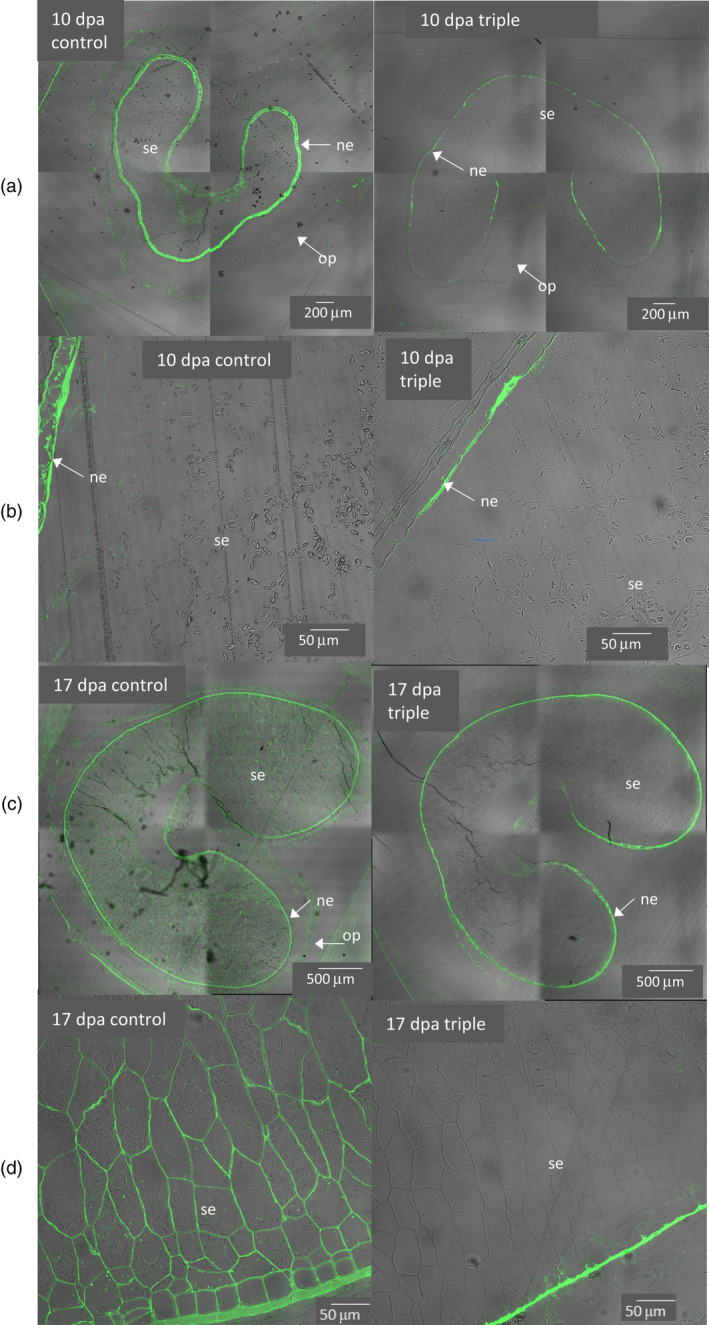
Overlay of fluorescence (green) and transmitted light (grey) images of sections from control and triple‐stack grain at 10 and 17 dpa. Fluorescence is due to LM11 immunolabelling of low substituted AX. Starchy endosperm (se), nucellar epidermis (ne) and outer pericarp (op) are indicated.

We made transverse grain sections at 10 and 17 days postanthesis (dpa) from three replicate plants of triple‐stack and null‐segregant control lines and used the LM11 monoclonal antibody which was raised against an unsubstituted penta‐β‐1,4‐xylanoside glycoprotein (McCartney *et al.*, 2005) and recognizes unsubstituted regions on AX but does not bind to highly substituted AX. We previously showed that immunolabelling with LM11 of grain sections was decreased in the TaIRX9b RNAi lines, explicable by the greater level of Araf substitution in the remaining AX (Lovegrove *et al.*, 2013), and we observed the same trends here (Figure 4; further examples Figure S3). At 10 dpa LM11 labelling is mostly confined to the nucellar epidermis and crease region in controls and this was virtually abolished in triple‐stack (Figure 4a) except for residual labelling occurring on the inner periclinal cell walls of nucellar epidermis (Figure 4b). In the triple‐stack we observed almost no labelling in endosperm and aleurone cells at 17 dpa (Figure 4c) with only the crushed nucellar epidermis still showing strong labelling (Figure 4d). Images in Figure 4 are overlays of confocal fluorescence and transmitted light which show unlabelled cell walls in grey; from these representative images and others, we observed that cell size and shape in all grain tissues were normal in the triple mutants although there was evidence that cell wall width of endosperm cells was decreased (most clearly visible in no‐antibody control images; Figure S4), as was also the case for TaIRX9b RNAi lines (Lovegrove *et al.*, 2013).

### Transcriptome from triple‐stack and control grain suggest pleiotropic effects to maintain cell wall integrity

**Figure 5:**
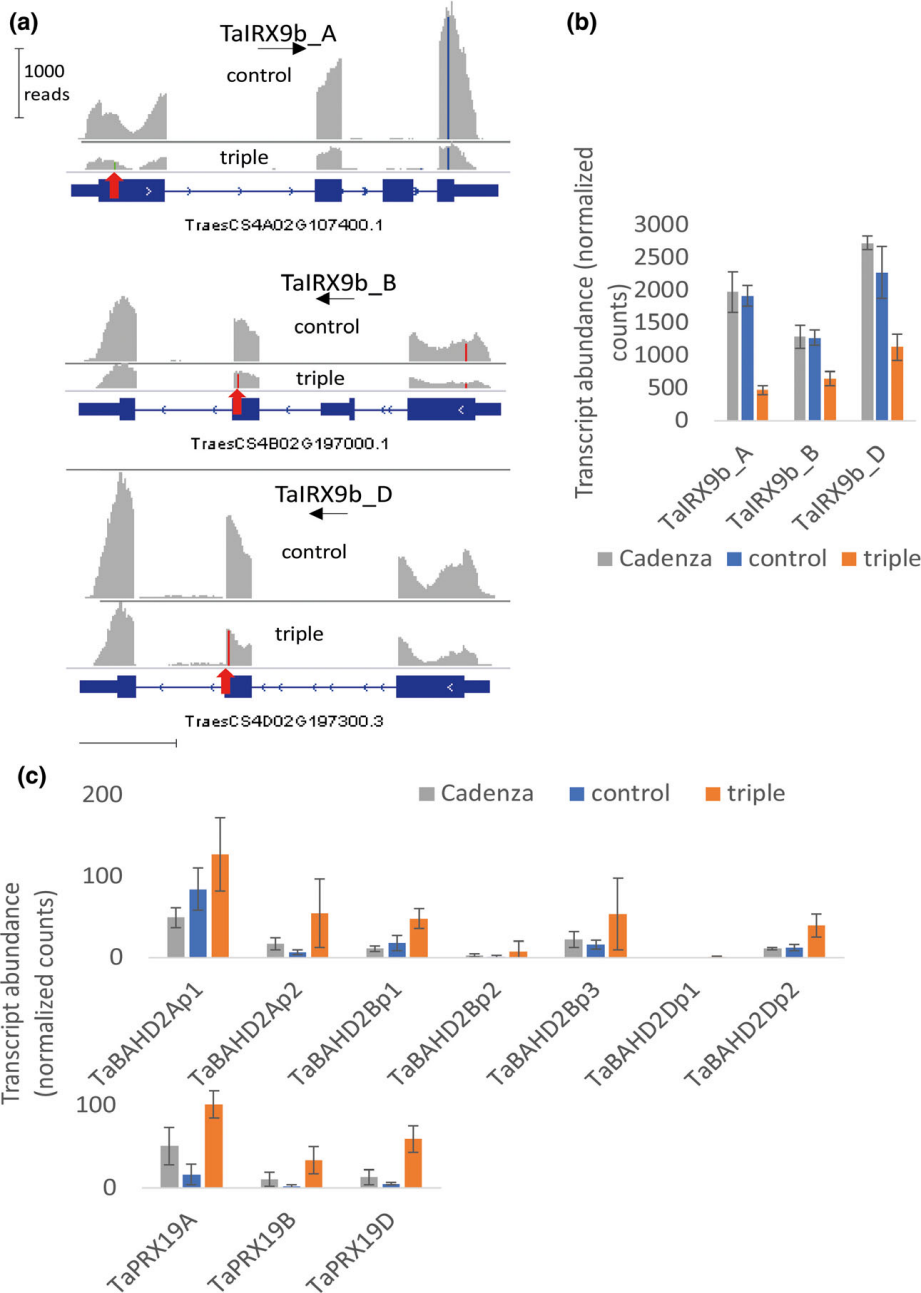
Transcript abundance of selected genes from RNAseq of endosperm tissue isolated at 21 dpa from Cadenza wild‐type, null‐segregant control and triple‐stack of TaIRX9b KO alleles. (a) Example read coverage of TaIRX9b genes. SNP differences from IWGSC Chinese Spring reference are coloured with the SNP conferring premature stop codon in triple indicated by red arrow. (b, c) Mean transcript reads per million mapped reads (TPM) ± SD (*n* = 3). (b) TaIRX9b genes (c) TaBAHD02 genes are TraesCS3A02G119500, TraesCS3A02G119700, TraesCS3B02G138900, TraesCS3B02G139000, TraesCS3B02G139100, TraesCS3D02G121700, TraesCS3D02G121800 and TaPRX19 genes are TraesCS3A02G325100, TraesCS3B02G354000, TraesCS3D02G318500, left to right.

We analysed the RNAseq transcriptome of endosperm and grain outer tissues from triple‐stack, control null‐segregant and wild‐type Cadenza lines isolated at 21 dpa. This stage was selected to be within grain‐fill period but still with high levels of transcription in the endosperm likely to influence properties of mature grain (Pellny *et al.*, 2012). Viewing mapped reads from control and triple mutants for the TaIRX9b genes confirms the introduced SNPs in all the triple‐stack samples (Figure S2). It also shows lower read count in the triple‐stack (Figure S2; Figure 5a); this is expected when a mutation moves the stop codon before the last exon (as is the case here for all three homeoalleles) as this triggers nonsense‐mediated decay (Gutierrez *et al.*, 1999). This phenomenon was presumably responsible for significantly lower TaIRX9b transcript abundance in endosperm (Figure 5b) in triple‐stack compared to control and Cadenza; in another study which targeted different genes from the same wheat population (Botticella *et al.*, 2018), expression of mutated target genes was also decreased.

We assessed other changes on the transcriptome by identifying all differentially expressed genes (DEGs) between the control and triple‐stack (*P* < 0.05, adjusted for multiple testing). Both control and triple‐stack lines would have many background mutations; discounting DEGs that also occurred as DEGs between Cadenza and control (likely to be non‐specific responses) and TaIRX9b themselves, there are 355 and 394 DEGs in endosperm and grain outer tissues, respectively (Tables S3 and S4 for endosperm and outer layers respectively) out of the 108 035 high confidence genes annotated in the IWGSC 1.1 wheat genome (Appels *et al.*, 2018). Contained within these sets are many transcripts associated with cell walls, suggestive of pleiotropic effects due to the loss of TaIRX9b function. For example, five expansin genes are down‐regulated in endosperm (Table S3), consistent with a cell wall integrity sensing mechanism responding to a loosening (Voxeur and Höfte, 2016) caused by loss of AX chains. Since we had previously observed that diferulate (diFA) levels were maintained despite loss of AX in endosperm of RNAi lines (Freeman *et al.*, 2017), we were interested to find a BAHD gene (TaBAHD2A) and a peroxidase gene (TaPRX19D) among the most up‐regulated within endosperm DEGs. BAHD genes in this clade are putatively involved in addition of FA (de Souza *et al.*, 2018) or *p*‐coumarate (pCA) to AX (Bartley *et al.*, 2013); we have recently demonstrated that the orthologs of these TaBAHD02 genes are dramatically up‐regulated by methyl‐jasmonate in Brachypodium accompanied by a rise in both FA and pCA linked to AX (Hyde *et al.*, 2018). Tandemly arranged TaBAHD02 paralogues are present on all subgenomes in wheat and were significantly up‐regulated in the endosperm of triple mutants (Figure 5c). TaPRX19D is from the family of class III peroxidases, members of which are involved in oxidative cross‐linking of cell wall proteins in dicots (Voxeur and Höfte, 2016) and FA in grasses (Burr and Fry, 2009); the homeologues of this gene are also up‐regulated in the endosperm of triple mutants (Figure 5c). Some other DEGs in endosperm and outer layers are also potentially related to xylan synthesis and remodelling (annotated in Tables S3 and S4); the most up‐regulated gene in endosperm being TraesCS6B02G187200 from the Trichome birefringence‐like family (Table S3). Members of this family are responsible for xylan acetylation (Xiong *et al.*, 2013) and intense acetylation of wheat arabinoxylan has been reported in developing grain although AX in mature grain is not acetylated (Veličković *et al.*, 2014).

### Cell wall ferulate composition of triple mutants shows increased dimerization

To decrease the number of background mutations, we undertook four rounds of backcrossing, selfed progeny and identified homozygous plants for the stacks and control (control = no TaIRX9b mutant alleles) lines. All results we present below are from these BC4F2 lines. We observed a decrease in cell wall ferulate monomer (FA) but an increase in diFA in mature grain from triple mutants compared to controls (Table 1). This increased dimerization was also significant in the endosperm fraction (Table 1). We previously observed similarly increased FA dimerization in endosperm cell walls in transgenic wheat where TaIRX9b genes were repressed by RNAi (Freeman *et al.*, 2017).

**Table 1:** Bound hydroxycinnamate composition of whole grain and endosperm (white flour) from control and triple‐stack lines. Averages ± SD, *n* = 3

	*p*CA	FA	diF 8‐5	diF 5‐5	diF 8‐O‐4	diF 8‐5BF	TOT diFA	FA dimerization*
Whole grain	[µg/g dwt]							
Control	41.7 ± 7.7	1352.2 ± 47.2	57.7 ± 3.0	43.9 ± 1.9	56.9 ± 4.1	70.2 ± 5.4	228.8 ± 9.6	0.14 ± 0.00
Triple	37.9 ± 4.9	986.8 ± 18.9	67.6 ± 2.1	51.6 ± 3.6	60.5 ± 6.7	85.8 ± 2.6	265.6 ± 13.1	0.21 ± 0.01
Triple/control	91%	73%	117%	117%	106%	122%	116%	147%
*P*‐value (*t*‐test)	0.508	0.000	0.010	0.030	0.471	0.011	0.017	0.001
Endosperm	[µg/g dwt]							
Control	18.4 ± 11.4	235.6 ± 96.1	11.8 ± 4.1	11.7 ± 4.5	18.2 ± 4.6	21.1 ± 7.9	62.7 ± 21.0	0.21 ± 0.02
Triple	10.3 ± 4.2	130.0 ± 18.4	9.5 ± 1.6	9.0 ± 0.9	14.1 ± 1.5	17.2 ± 3.1	49.8 ± 6.9	0.28 ± 0.00
Triple/control	56%	55%	81%	77%	77%	82%	79%	130%
*P*‐value (*t*‐test)	0.309	0.135	0.427	0.367	0.214	0.471	0.369	0.003

* FA dimerization = diFA/(diFA + FA).

### Viscosity of grain extracts from triple‐stack and control lines is decreased due to loss of long‐chain WE‐AX

**Figure 6:**
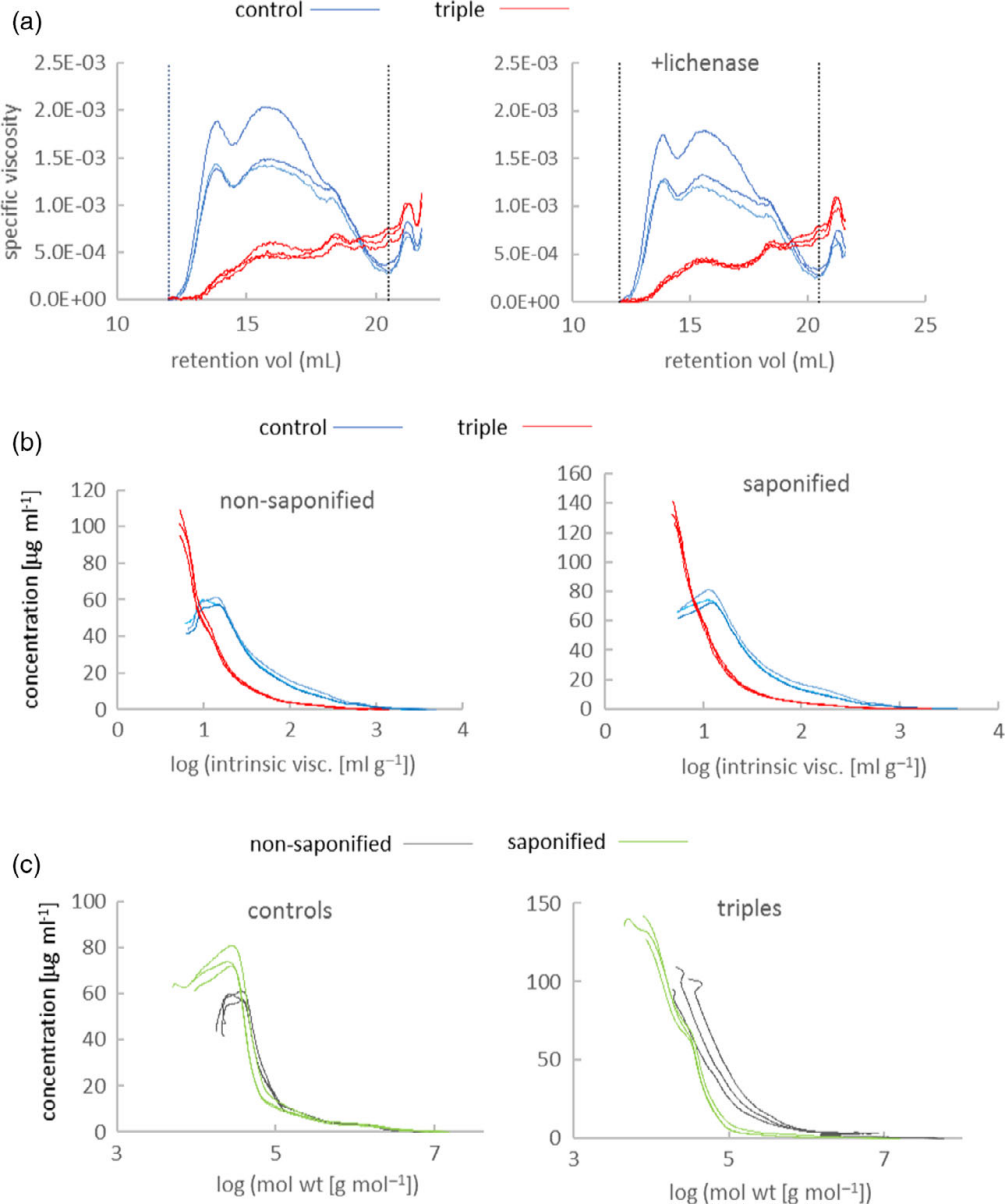
SE‐HPLC analyses on extracts from control and triple‐stack grain showing data from three independent biological reps of each. (a) Chromatograms showing specific viscosity of samples with and without lichenase treatment. Vertical dotted lines denote integration limits used. (b) Distributions of log intrinsic viscosity from lichenase‐treated extracts with or without saponification treatment. (c) Distributions of log molecular weight from lichenase‐treated extracts or without saponification treatment.

Viscosity of water extracts from wholemeal flour is primarily determined by WE‐AX with smaller contributions from soluble (1,3;1,4)‐beta‐glucan and low molecular weight molecules such as arabinogalactan peptide (AGP) and raffinose. These extract viscosities show considerable variation between samples of wild‐type wheat (Freeman *et al.*, 2016) and the controls in this experiment were at the lower end of the range; despite this, we found that specific viscosity in triples was about 45% of that in controls (Table 2). As expected, we found decreased Xyl and Ara content in the extracts (Table 2); Ara was decreased by less indicating increased Ara substitution in WE‐AX as found previously in transgenics (Lovegrove *et al.*, 2013) even allowing for Ara from AGP which will also be present. We examined the size‐exclusion HPLC (SE‐HPLC) profiles of extracts from grain of triple‐stack and control lines (Figure 6) and found that the contribution of high molecular weight molecules to viscosity was proportionately more decreased in triple mutants (Fig 6a). The part of the profile between vertical lines in Figure 6a correspond to viscosity contributed only by large WE‐AX and (1,3;1,4)‐beta‐glucan molecules. The integrals of viscosity between these limits account for about 65% of bulk extract viscosities given in Table 2, the remainder can be attributed to low molecular weight WE‐AX and other small molecules with long retention times. We treated samples with lichenase to specifically remove high molecular weight (1,3;1,4)‐beta‐glucan (Figure 6a) which removed 0.01–0.02 contribution to integral of specific viscosity for all samples (average total integrals were 0.23 and 0.09 for controls and triples respectively). This suggests that amounts of high molecular weight (1,3;1,4)‐beta‐glucan were similar in extracts from control and triple grain. The lichenase‐insensitive contribution to viscosity from molecules between the retention time limits is the product of the amount and intrinsic viscosity of WE‐AX. Intrinsic viscosity of WE‐AX is determined mostly by chain length (Dervilly‐Pinel *et al.*, 2001), although some effect of diFA mediated cross‐linking has been reported (Dervilly *et al.*, 2000). We found distributions of intrinsic viscosity to be not much affected by saponification and consequent loss of diFA in our samples (Figure 6b); average intrinsic viscosities (area under non‐log version of curves) for non‐saponified and saponified were respectively 53 and 49 mL/g for control and 15 and 13 mL/g for triple‐stack samples. We found much lower concentrations of all WE‐AX molecules with log intrinsic viscosity >1 in triple mutants compared to controls and no WE‐AX with log intrinsic viscosity >2.8 in triples. Using linear relationship between log chain length and log intrinsic viscosity derived for non‐feruloylated WE‐AX (Dervilly‐Pinel *et al.*, 2001) this corresponds to amounts of all WE‐AX molecules >10 Xyl length being decreased in triple mutants and chains of >2000 being undetectable.

**Table 2:** Monosaccharide composition (excluding Glc) and viscosity of water extracts from grain of control and triple mutant lines. Averages ± SD, *n* = 3

	Monosaccharide [mg/g dwt]				Specific viscosity
	Xyl	Ara	Gal	Man	
Control	3.75 ± 0.15	2.93 ± 0.09	2.83 ± 0.04	0.17 ± 0.04	0.36 ± 0.07
Triple	1.33 ± 0.08	1.94 ± 0.07	3.25 ± 0.01	0.16 ± 0.03	0.17 ± 0.01
Triple/control	35%	66%	115%	92%	46%
*P*‐value *t*‐test	1.E−05	0.0001	4.E−05	0.6559	0.0068

### Grain WE‐AX has more diFA and is more cross‐linked in triple‐stack lines

We found that WE‐AX from triples had 2.0‐fold higher FA content and 2.6‐fold higher diFA content per unit xylose than controls (Table 3). We investigated whether this increased amount of diFA was associated with increased inter‐molecular cross‐linking of WE‐AX by examining effect of saponification (which removes FA and diFA) on WE‐AX molecular weight (MWt) distribution estimated by multi‐angle laser light scattering on SE‐HPLC (Figure 6c). We found that saponification decreased amounts of higher MWt WE‐AX molecules in both controls and triples but the decrease was much greater in triples (Figure 6c). The effect of saponification on average MWt (area under non‐log version of curves in Figure 6c) was a 6‐fold decrease for triples and a 1.2‐fold decrease for controls; this suggests most WE‐AX molecules from triples are cross‐linked via diFA bridges to multiple others whereas such cross‐linking is comparatively rare in WE‐AX from controls.

**Table 3:** Bound hydroxycinnamate composition of water extracts from grain of control and triple‐stack. Two forms of diFA were detected; 8‐O‐4′ diFA and 8‐5′benzofuran (BF). The sum of these is given in diFA column. Averages ± SD, n = 3

	Per unit Xyl µg/mg Xyl			8‐5′BF	diFA
	pCA	FA	8‐O‐4′		
Control	0.15 ± 0.06	6.14 ± 0.76	0.14 ± 0.00	0.20 ± 0.01	0.35 ± 0.01
Triple	0.38 ± 0.05	12.47 ± 0.78	0.47 ± 0.04	0.43 ± 0.06	0.90 ± 0.10
Triple/control	257%	203%	330%	209%	259%
*P*‐value *t*‐test	8.0E−03	5.5E−04	1.5E−04	2.9E−03	5.8E−04

## Discussion

We have demonstrated that the three homeologous GT43_2 genes that are highly expressed in endosperm (Figure 1) are functional orthologues of IRX9 in Arabidopsis (Figure 2) so name them TaIRX9b. This is a noteworthy finding since the Arabidopsis IRX9 is involved in synthesis of secondary cell wall xylan which carries glucuronosyl and acetyl decorations whereas wheat endosperm AX is decorated only with arabinofuranosyl residues.

By identifying and stacking loss‐of‐function alleles of TaIRX9b from a mutagenized wheat population, we were able to produce lines with decreased AX in the grain. The lack of a strong effect on AX in double mutants suggested that each homoelogue can support near‐wild‐type levels of AX biosynthesis; thus, only the triple‐stack shows a substantial reduction. (Figure 3). Microscopy showed abolition of LM11 immunolabelling in triple mutants but no change in cell shape (Figure 4) which is consistent with other observations that show large effects of abolition of TaIRX9b function on grain AX traits but no obvious effects on morphology.

We found a large decrease in viscosity of extracts from grain of triple mutants (Table 2); low extract viscosity is a potentially valuable trait for use of wheat in alcohol production and animal feed. Using mutations avoids the problems of potential transgene silencing and regulatory restrictions of GM wheat lines. Furthermore, we observed no change in grain weight (Figure 3c), a key quality parameter for most uses. These lines therefore offer potential for development into wheat varieties intended for these applications.

The decrease in extract viscosity in triple mutants was entirely explained by a decrease in amount of long‐chain WE‐AX (Figure 6a,b). A substantial decrease in amount of long‐chain AX might be expected to weaken the starchy endosperm cell walls where it accounts for 65% of the polysaccharide and we found evidence that there were adaptive changes to compensate for such a weakening. In the transcriptome of the developing grain of controls and triple mutants, we found some of the most strongly differentially regulated genes are associated with cell wall synthesis or remodelling, particularly up‐regulation of BAHD02 and peroxidase genes (Figure 5c); the BAHD02 genes are from a clade containing genes thought to be responsible for the addition of FA and pCA residues to AX (Bartley *et al.*, 2013; Mitchell *et al.*, 2007; de Souza *et al.*, 2018) and peroxidases in the apoplast are believed to be required for oxidative dimerization of FA on AX (Burr and Fry, 2009). Therefore, the up‐regulation of BAHD02 and peroxidase genes may be responsible for the increased FA per unit AX and FA dimerization that we observed here (Tables 1 and 3) and previously (Freeman *et al.*, 2017) in response to TaIRX9b repression. Brachypodium orthologues of TaBAHD02 and multiple peroxidases were also substantially up‐regulated following methyl‐jasmonate treatment which also induced increases in cell wall pCA, FA and FA dimerization (Hyde *et al.*, 2018). Here, we obtained evidence that the increased amount of diFA per unit AX resulted in greater cross‐linking between WE‐AX molecules from triples, shown by the much greater decrease in molecular weight induced by saponification (Figure 6c).

Such compensatory effects are consistent with knowledge of other primary cell walls, where extreme perturbations can be completely compensated for by changes in composition and structure that are induced by sophisticated sensing of cell wall integrity, although little is known about how this operates in grass primary cell walls (Voxeur and Höfte, 2016). Some of the other DEGs within the endosperm transcriptome from the triples (Table S3) may also reflect some of these cell wall adaptive changes, for example down‐regulation of expansins and some aspects of the signalling steps involved (kinases and calcium‐binding proteins). Our RNAseq transcriptomes therefore represent a resource that can be exploited to formulate hypotheses on control of wheat grain cell walls.

We have demonstrated that abolishing TaIRX9b function has a large effect on long‐chain WE‐AX from grain, but morphology and cell shape are unchanged. Assuming all xylan synthesis requires IRX9 components within xylan synthase complexes, the remaining xylan in the triples is presumably synthesized with IRX9 components encoded by some of the 7 other triads resembling IRX9 (Figure 1). We do not know whether the specific role of TaIRX9b genes in synthesizing long‐chain WE‐AX is due only to their expression pattern, which will determine the context of the encoded IRX9 protein (e.g. other proteins and metabolites present), or whether the sequence of the encoded IRX9 also confers specialist properties, for example leading to longer chains. Complementation experiments of these triple‐stack lines with different IRX9 genes could be used to address this.

The apparent lack of effect of knocking out TaIRX9b function apart from on grain AX raises questions why these genes were selected for during evolution of wheat ancestors. WE‐AX acts as an antinutritive factor in wheat grain consumed by poultry, possibly due to its effect on viscosity (Fincher and Stone, 1986). We speculate that WE‐AX is present mostly as an antinutritive factor and that having separate IRX9 genes responsible for WE‐AX allows for separate control of this (e.g. expression largely confined to grain during grain‐filling) as opposed to xylans essential for cell wall function. Therefore, abolition of TaIRX9b function impacts greatly on viscosity of extracts from grain with little effect on cell wall integrity, although in the case of endosperm cell walls this appears to require compensatory mechanisms.

In conclusion, building on our previously reported effects of suppressing homeologous GT43 genes by RNAi, we have now shown that this wheat GT43 gene is a true IRX9 capable of restoring xylan synthesis to *irx9* mutant, despite the major differences between wheat endosperm AX and xylan in secondary cell walls of Arabidopsis. We developed novel wheat lines lacking functional TaIRX9b by stacking three knockout homeoalleles. Using mutations avoids the problems of potential transgene silencing and regulatory restrictions of GM wheat lines. These lines therefore offer potential for development into wheat varieties intended for alcohol production and animal feed. They are also a resource for investigating the control of endosperm cell walls, as shown by the evidence of compensatory changes to maintain cell wall function, with mechanisms which may well apply more widely in primary cell walls of grasses.

## Materials and Methods

### Phylogenetic analysis

Peptide sequences encoded by GT43 genes were taken from loci listed in Table S1. Alignments and generation of maximum likelihood tree were performed as previously described (Pellny *et al.*, 2012; de Souza *et al.*, 2018).

### Complementation

Expression vectors were obtained using Golden Gate Modular Cloning (MolClo) with the standard parts (Patron *et al.*, 2015). *AtIRX9*, *TaIRX9_2* coding sequences and *IRX3* promoter domesticated DNA sequences where synthesized by GENEWIZ®. Expression vectors consisted in Level 2 construct containing in position R1 Oleosin‐GFP selection marker (Shimada *et al.*, 2010) and *pIRX3‐AtIRX9‐myc* or *pIRX3‐TaIRX9_2‐myc* in position R2. For complementation experiments, Arabidopsis *irx9.1* mutant plants were transformed using the standard floral dip method (Clough and Bent, 1998). Seven‐week‐old transgenic lines and the controls were used to evaluate growth complementation. Neutral monosaccharide composition of non‐cellulosic polysaccharides of basal stems was analysed by high‐performance anion‐exchange chromatography coupled to pulsed amperometric detector (HPAEC‐PAD) as described in Tryfona *et al. *(2014).

### Identification of mutant wheat lines

We screened lines from the mutagenized wheat population derived from cv. Cadenza (Rakszegi *et al.*, 2010), searching for mutations in TaIRX9b genes. Identification of mutations in target genes was by high‐resolution melt analysis (Botticella *et al.*, 2011) followed by Sanger sequencing of amplicons. The high mutation rate in this population means that there is a good chance of finding loss‐of‐function mutations for any target in relatively few lines and we found a total of 7 premature stop codons over all three homeologs of TaIRX9b after screening 2150 lines; we selected one of these for each homeolog (Figure S1). These alleles were followed in progeny of crosses using co‐dominant KASP markers (Limagrain genotyping facility, France) using primers given in Table S2 to identify homozygous plants. Lines carrying mutations in homeologs were crossed and selfed and F2 homozygotes identified to produce the aabbDD, aaBBdd and AAbbdd double stacks. The aabbDD stack was crossed with AABBdd, selfed and F2 homozygotes identified to produce the M5 triple‐stack and null‐segregant controls. The triple‐stack line was subjected to 4 rounds of backcrossing to Cadenza, at each generation selecting lines carrying all three mutations. The BC4F1 line was selfed and homozygotes selected to give BC4F2 triple mutants and null‐segregant controls.

### Plant sampling

Control and lines carrying mutant alleles were grown in small plots in the field at Rothamsted in 2016 (Figure 3) or in pots in a controlled temperature glasshouse with supplementary lighting (Figures 4, 5, 6). Results shown in Figures 3, 4, 5 are from M5 lines; results in Tables 1, 2, 3 and Figure 6 are from BC4F2 lines.

### RNAseq

Endosperms were separated from grain outer layers by rolling them out at 21 days postanthesis (dpa) from three biological replicates of each line (null‐segregant, triple mutant, non‐mutagenized Cadenza); RNA was isolated from both endosperm and outer layers giving 18 samples. Total RNA isolation and quality control was as in (Pellny *et al.*, 2012). Library preparation and sequencing were carried out by Oxford Genomics Centre, UK. Reads were trimmed to remove adapter and low‐quality ends and then mapped to the IWGSC refseq1.1 genome (Appels *et al.*, 2018) using the HISAT mapper with default settings (Kim *et al.*, 2015). The 18 libraries all generated between 17 and 26 million uniquely mapped reads. Expression was estimated using FeatureCounts (Liao *et al.*, 2013) with parameter set for forward reads only (since stranded libraries were used) and other parameters set to default. Differentially expressed genes were identified using DEseq2 software (Love *et al.*, 2014). All RNAseq read data and protocols are available under ArrayExpress accession E‐MTAB‐8237.

### Microscopy

Wheat grains were taken from the middle spikelets of the ear at 10 and 17 dpa. Wheat grains were fixed in 4% paraformaldehyde, 2.5% glutaraldehyde and dehydrated in a graded ethanol series. The samples were then processed through increasing concentrations of LR White Resin (Agar Scientific, Stansted, Essex, UK, R1281) and embedded at 58 °C for 16–20 h in a nitrogen‐rich environment. 1 µm sections of the resin blocks were cut with a Reichert‐Jung ultramicrotome, and dried onto Polysine‐coated slides (Agar Scientific, Stansted, Essex, UK, L4345) at 40 °C. Sections were immunolabelled as described by (Tosi *et al.*, 2011) using the primary antibody LM11 and Alexa Fluor 488 goat anti‐rabbit IgG (Invitrogen, Paisley, Renfrewshire, UK A11008). Images were acquired with a Zeiss, Cambourne, Cambridge, UK LSM 780 confocal microscope using Zeiss, Cambourne, Cambridge, UK ZEN 2010 software.

### Cell Wall hydroxycinnamic acid content

Bound hydroxycinnamic acids were extracted from AIR prepared from whole grain or white flour, or from water extracts, and quantified by HPLC using a UPLC Kinetex Phenyl‐Hexyl (150 mm × 4.6 mm, 5 µm) column as previously described (Freeman *et al.*, 2017).

### AX content, Monosaccharides, Viscometry and Size‐Exclusion HPLC

Total and WE‐AX content were determined as detailed in Finnie *et al.* (2006). Monosaccharide analysis following acid hydrolysis was as described by Bromley *et al. *(2013). Relative viscosity of water extracts was determined using the method of Freeman *et al. *(2016). WE extracts for use in SE‐HPLC were prepared as for relative viscosity measurements except that following centrifugation the supernatant was aliquoted (to give equivalent of 200 mg/mL starting material), made up to 1 mL with water and digested with recombinant lichenase as described in Freeman *et al. *(2017). Following boiling and centrifugation, aliquots of 950 µL of supernatants were removed and dried under vacuum. Samples for saponification were resuspended in 400 µL 2 m NaOH, vortexed for 30 s and incubated in the dark for 16 h at 40 °C. Following incubation, samples were neutralized by addition of 415 µL of 2 m HCL and vortexed. Control samples were resuspended in 815 µL of 2 m NaCl. 500 µL of control or saponified samples were desalted using PD Minitrap G‐25 columns (GE Healthcare, Amersham, Herts, UK) using the manufacturer's spin protocol. Samples were filtered through 0.45 µm PVDF filters and 100 µL injected onto the SE‐HPLC system which was as in Kristek *et al. *(2019) with the following modifications; the column temperature was set at 35 °C and running buffer contained 0·02% sodium azide and 0.1 m sodium nitrate. Size‐exclusion columns were Shodex OH‐Pak SB 806M HQ and SB 804 HQ columns in series, and a flow rate of 0.5 mL/min. Data were analysed using Wyatt, Haverhill, Suffolk, UK ASTRA software.

## Conflict of interest

The authors declare that a related patent has been granted.

## Author contributions

TKP and AP identified mutants with guidance from ALP; TKP produced the triple mutant lines and performed the RNAseq experiments; SB provided the genotyping; AJW, JF, KH, AP and OK performed the other experiments on wheat; HT and JDC performed Arabidopsis experiments; RACM, TKP, AL, ALP and PD provided supervision of experiments; and RACM conceived the project, analysed the results and wrote the article with contributions from TKP, JF, HT, PD, PRS, AL and ALP.

## Supporting information


**Figure S1** Alignment of TaGT43_2 (TaIRX9b) sequences showing mutations used.

**Figure S2** Confirmation of TaIRX9b genotype from RNAseq in controls and triple‐stack lines.

**Figure S3** Further example images as in Figure 4.

**Figure S4** No‐antibody control images for immunolabelling of grain sections of control and triple‐stack lines.

**Table S1** GT43 gene loci from Arabidopsis, rice and wheat.

**Table S2** Primers and conditions for competitive PCR assay marker.

**Table S3** DEGs identified from endosperm RNAseq.

**Table S4** DEGs identified from pericarp RNAseq.
