# Supplementary material for: Loss of TaIRX9b gene function in wheat decreases chain length and amount of arabinoxylan in grain but increases cross‐linking
Source: Plant Biotechnol J. 2020 May 17;18(11):2316–27. doi: 10.1111/pbi.13393 (PMC7589350; doi:10.1111/pbi.13393)
Supplement: Supplementary file 1 — Figure S1 Alignment of TaGT43_2 (TaIRX9b) sequences showing mutations used. [file PBI-18-2316-s007.pptx]

## Slide 1
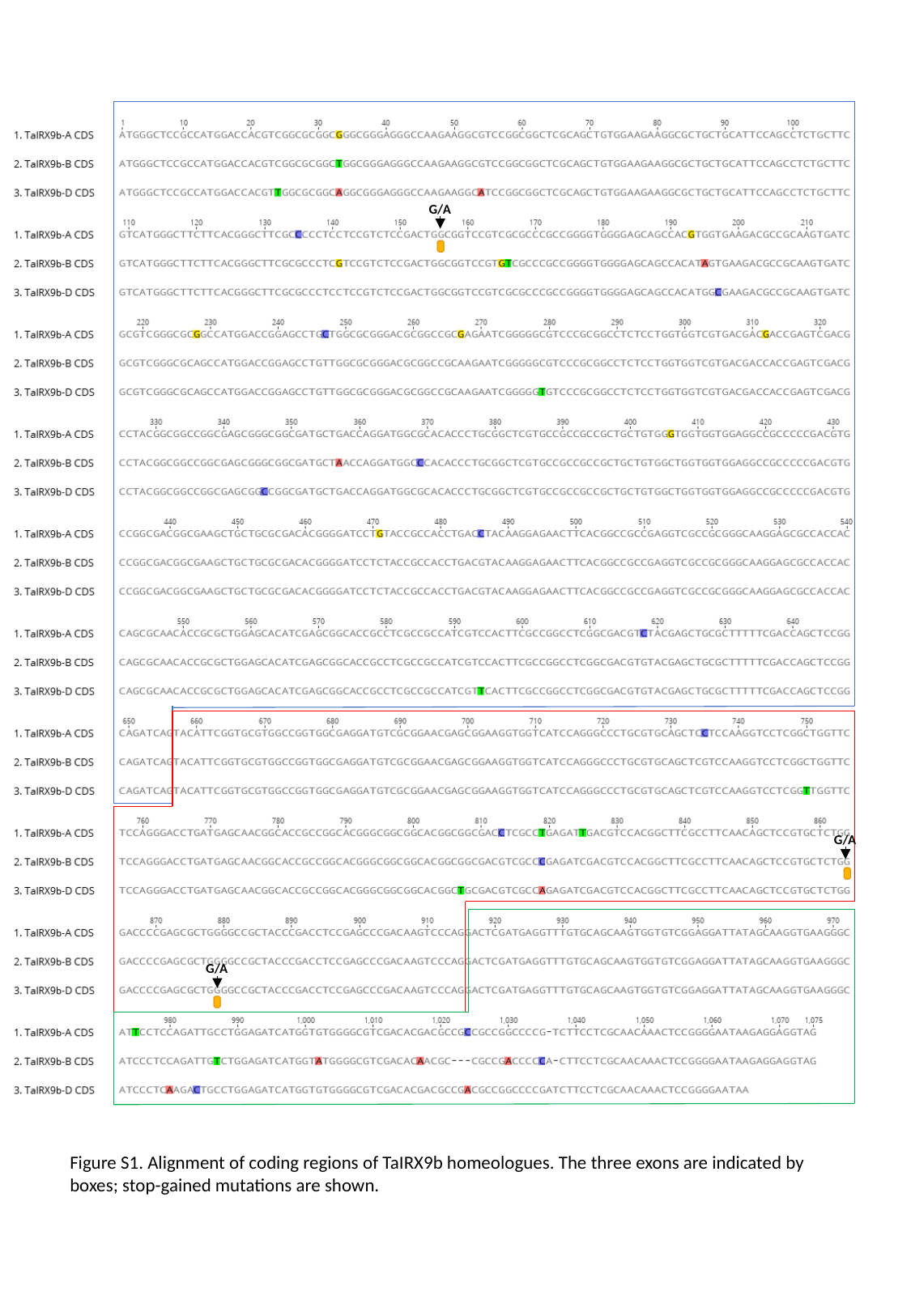

G/A
G/A
G/A
Figure S1. Alignment of coding regions of TaIRX9b homeologues. The three exons are indicated by boxes; stop-gained mutations are shown.
