# Supplementary material for: Loss of TaIRX9b gene function in wheat decreases chain length and amount of arabinoxylan in grain but increases cross‐linking
Source: Plant Biotechnol J. 2020 May 17;18(11):2316–27. doi: 10.1111/pbi.13393 (PMC7589350; doi:10.1111/pbi.13393)
Supplement: Supplementary file 2 — Figure S2 Confirmation of TaIRX9b genotype from RNAseq in controls and triple‐stack lines. [file PBI-18-2316-s006.pptx]

## Slide 1
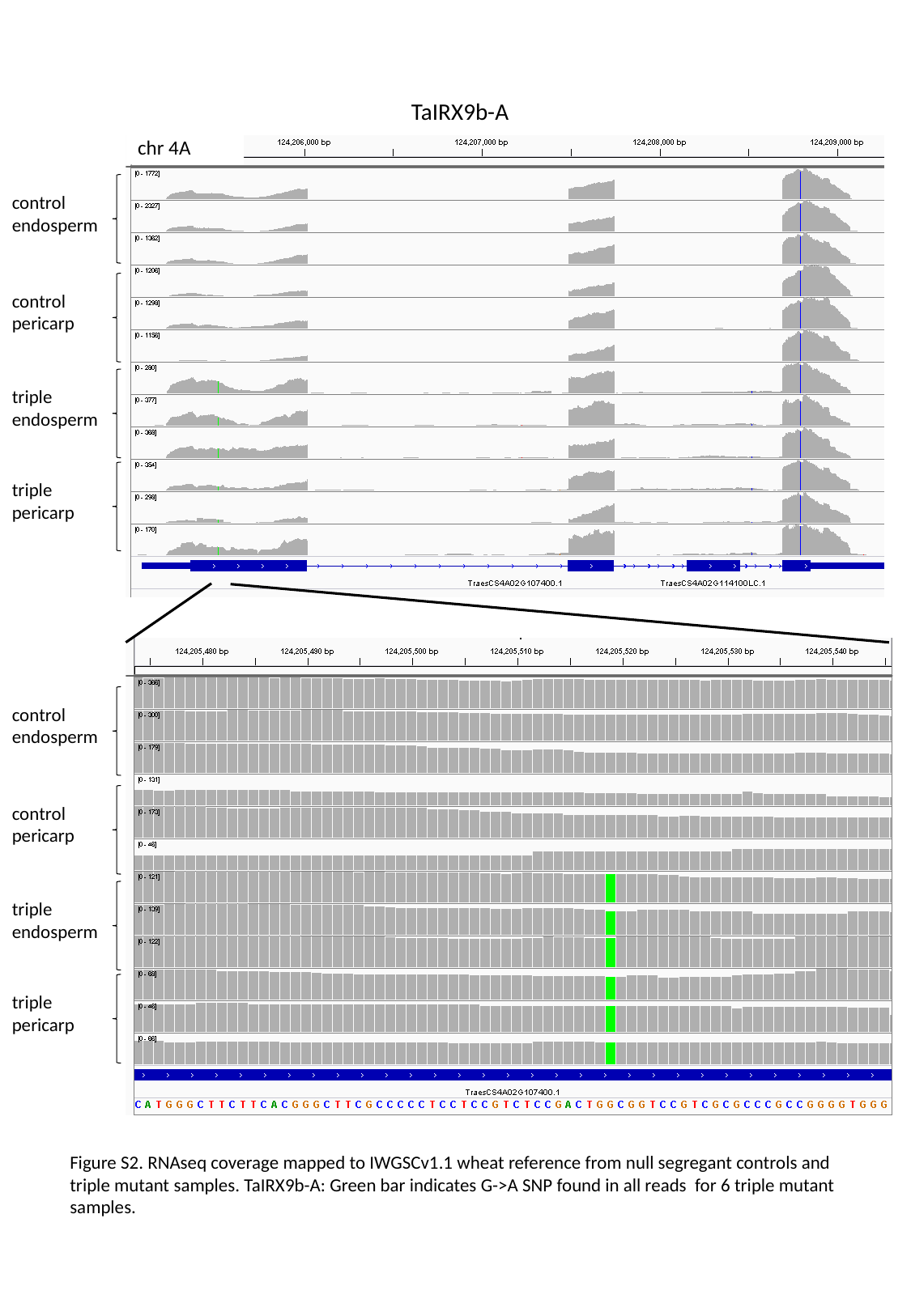

TaIRX9b-A
chr 4A
control endosperm
control pericarp
triple endosperm
triple pericarp
control endosperm
control pericarp
triple endosperm
triple pericarp
Figure S2. RNAseq coverage mapped to IWGSCv1.1 wheat reference from null segregant controls and triple mutant samples. TaIRX9b-A: Green bar indicates G->A SNP found in all reads for 6 triple mutant samples.

## Slide 2
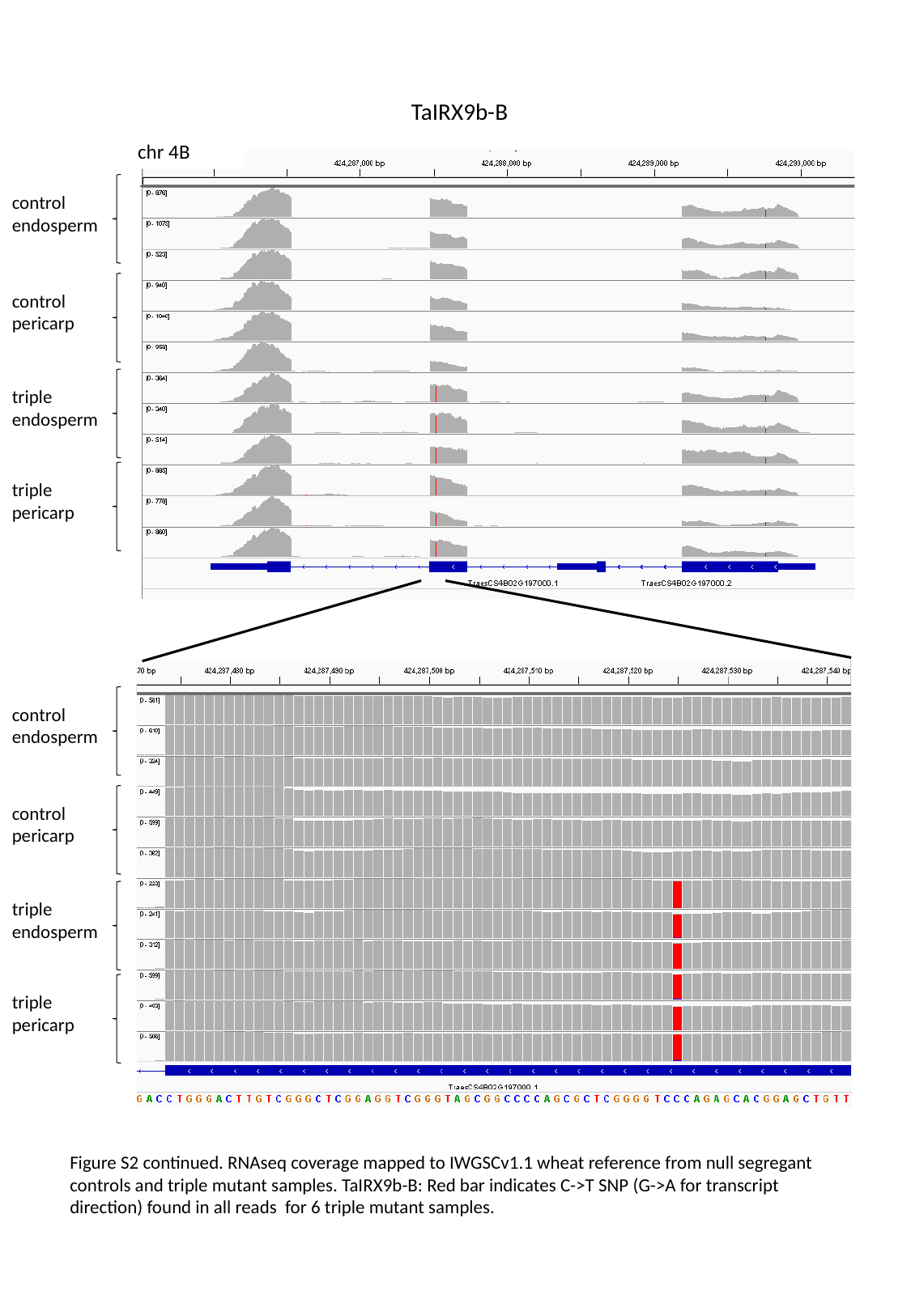

TaIRX9b-B
chr 4B
control endosperm
control pericarp
triple endosperm
triple pericarp
control endosperm
control pericarp
triple endosperm
triple pericarp
Figure S2 continued. RNAseq coverage mapped to IWGSCv1.1 wheat reference from null segregant controls and triple mutant samples. TaIRX9b-B: Red bar indicates C->T SNP (G->A for transcript direction) found in all reads for 6 triple mutant samples.

## Slide 3
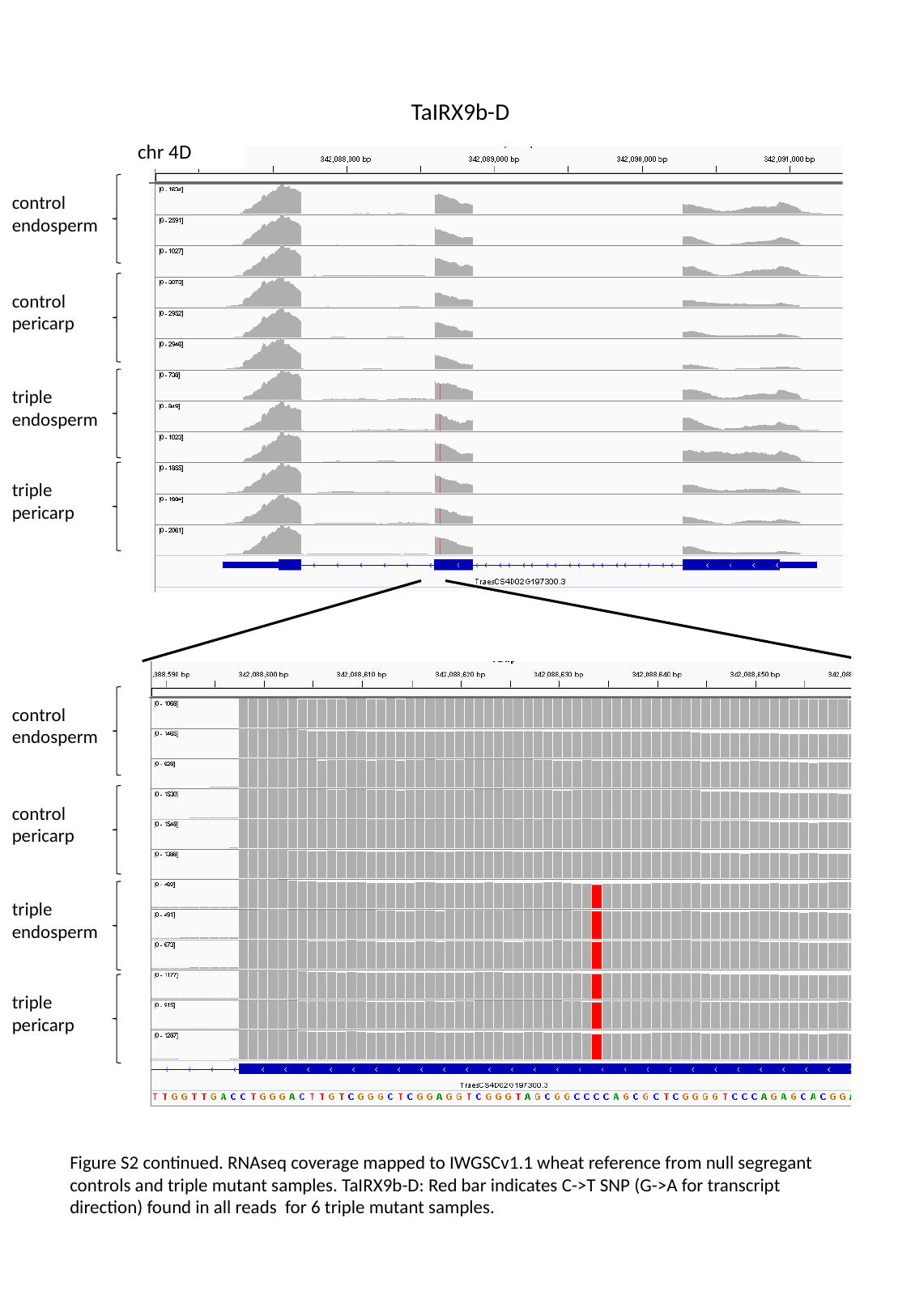

TaIRX9b-D
chr 4D
control endosperm
control pericarp
triple endosperm
triple pericarp
control endosperm
control pericarp
triple endosperm
triple pericarp
Figure S2 continued. RNAseq coverage mapped to IWGSCv1.1 wheat reference from null segregant controls and triple mutant samples. TaIRX9b-D: Red bar indicates C->T SNP (G->A for transcript direction) found in all reads for 6 triple mutant samples.
