# Supplementary figures and images for: Loss of TaIRX9b gene function in wheat decreases chain length and amount of arabinoxylan in grain but increases cross‐linking
Source: Plant Biotechnol J. 2020 May 17;18(11):2316–27. doi: 10.1111/pbi.13393 (PMC7589350; doi:10.1111/pbi.13393)

## Slide 1
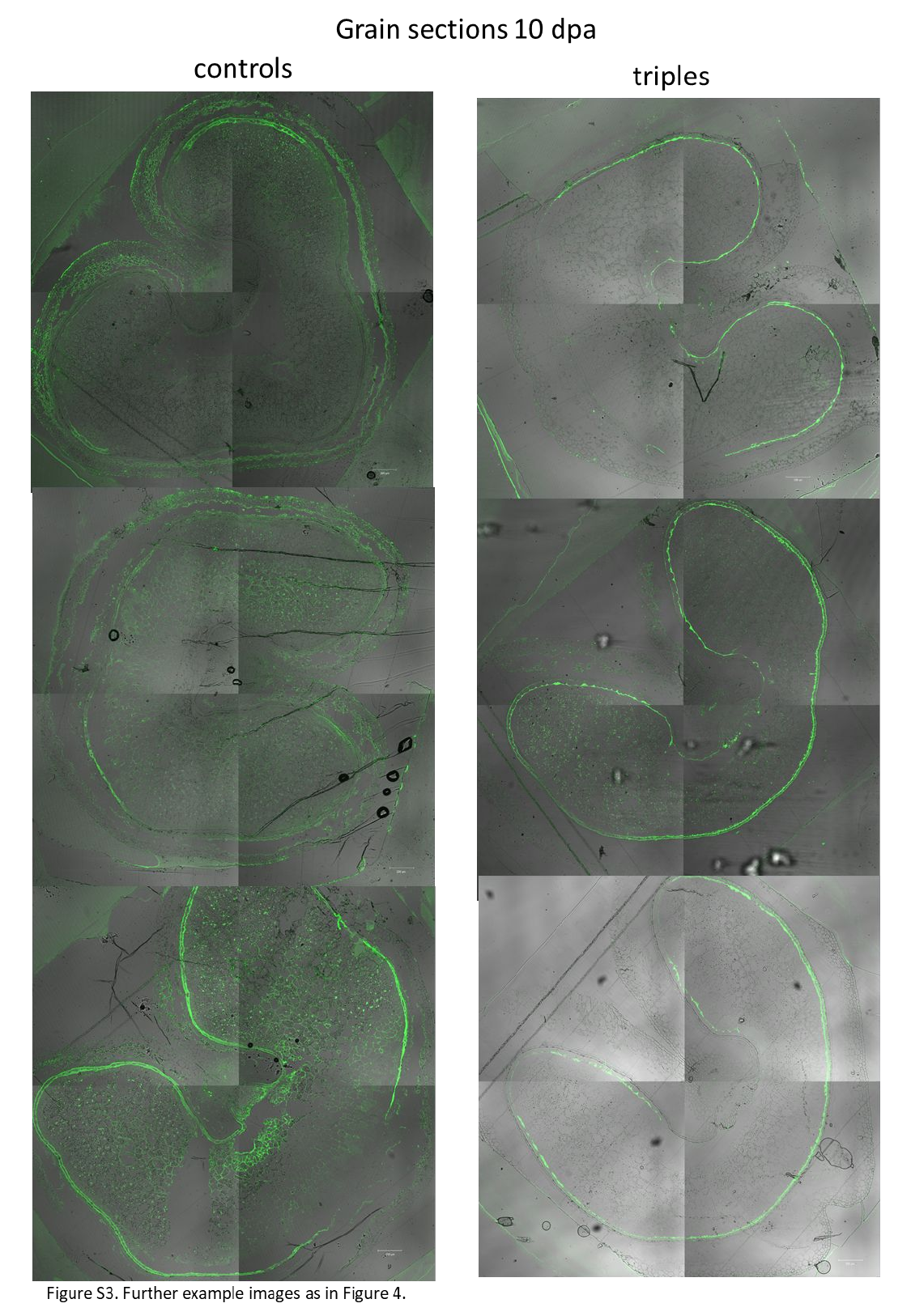

## Slide 2
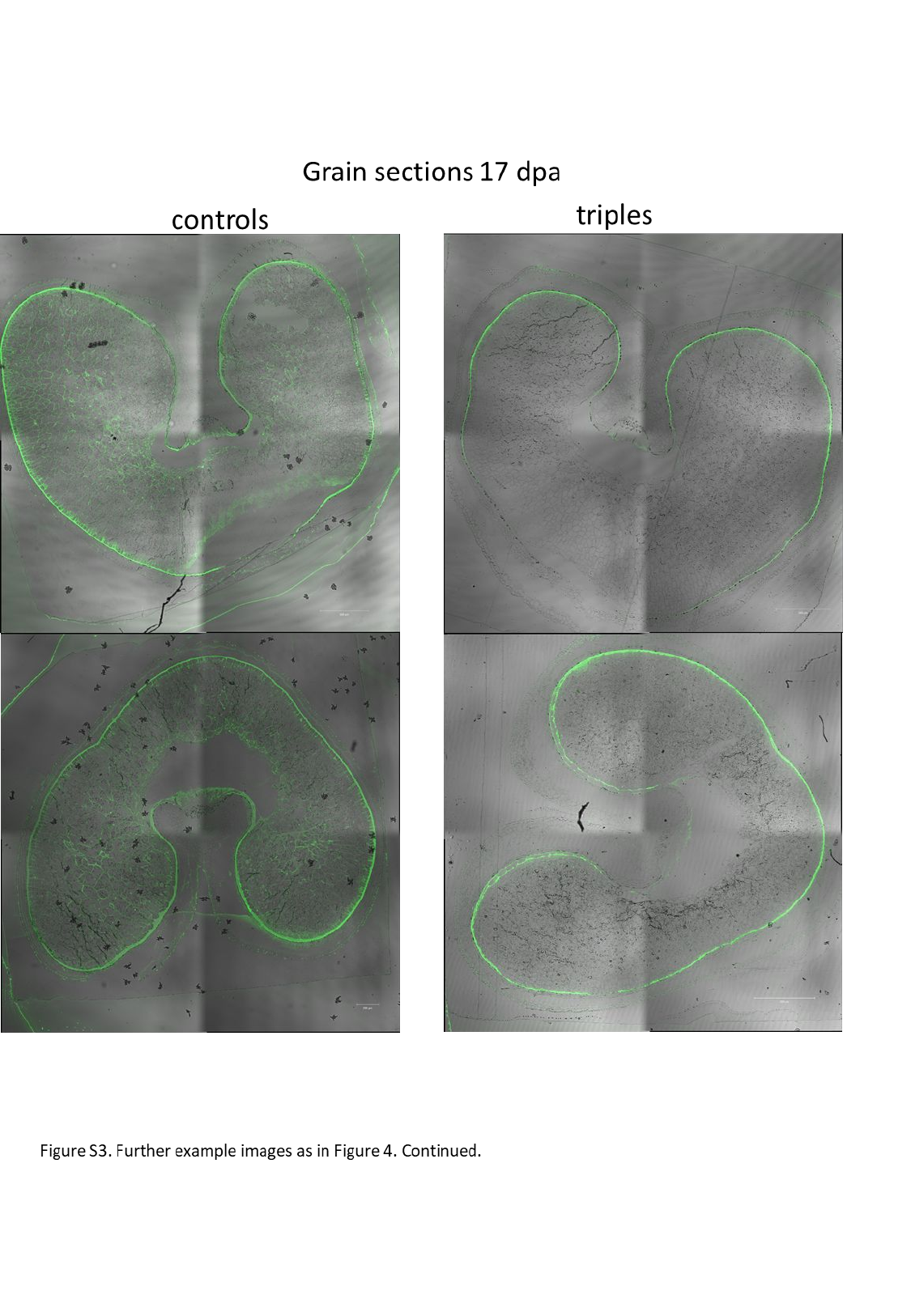

Supplement: Supplementary file 3 — Figure S3 Further example images as in Figure 4. [file PBI-18-2316-s005.pptx]
