# Supplementary material for: Loss of TaIRX9b gene function in wheat decreases chain length and amount of arabinoxylan in grain but increases cross‐linking
Source: Plant Biotechnol J. 2020 May 17;18(11):2316–27. doi: 10.1111/pbi.13393 (PMC7589350; doi:10.1111/pbi.13393)
Supplement: Supplementary file 4 — Figure S4 No‐antibody control images for immunolabelling of grain sections of control and triple‐stack lines. [file PBI-18-2316-s008.pptx]

## Slide 1
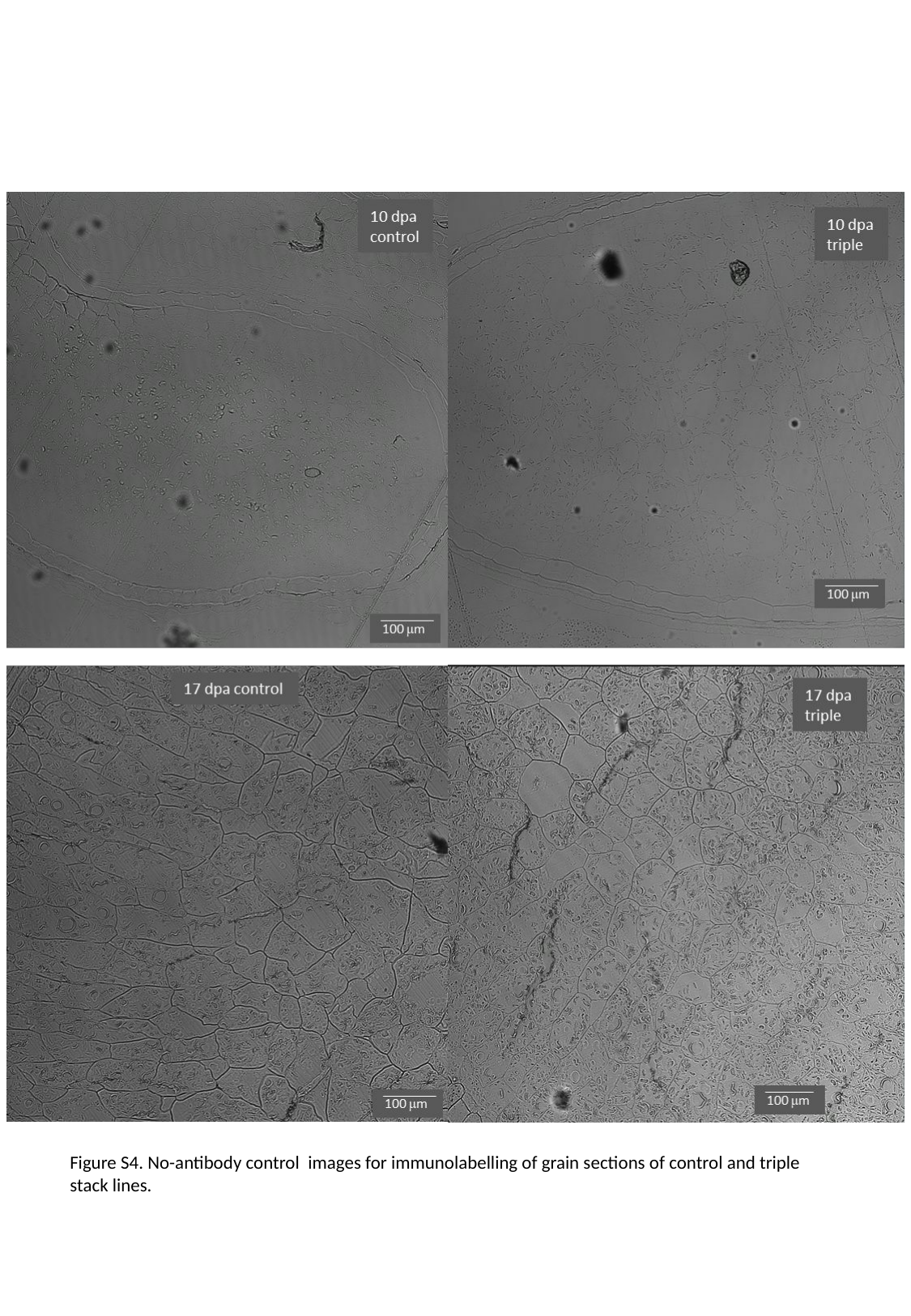

Figure S4. No-antibody control images for immunolabelling of grain sections of control and triple stack lines.
